# Combined use of intravenous and topical tranexamic acid efficiently reduces blood loss in patients aged over 60 operated with a 2-level lumbar fusion

**DOI:** 10.1186/s13018-020-01758-8

**Published:** 2020-08-20

**Authors:** Jianjiang Li, Long Wang, Tao Bai, Yanlu Liu, Yifei Huang

**Affiliations:** Department of Orthopedic Surgery, Traditional Chinese Medicine Hospital of Xinjiang Medical University, Urumqi, Xinjiang, 830000 China

**Keywords:** Tranexamic acid, Total hip arthroplasty, Blood loss, Combined treatment

## Abstract

**Purpose:**

The current study was conducted to assess the efficacy and safety of the intravenous (IV) administration combined with topical administration of tranexamic acid (TXA)in patients (aged over 60) scheduled for a 2-level lumbar fusion surgery.

**Methods:**

Two hundred eighty patients scheduled for a 2-level lumbar fusion surgery were randomized into four groups, including an IV group, a local group, a combined group, and a control group. Patients in the combined group, in the IV group, in the topical group, and in the control group were administrated with 15 mg/kg of IV-TXA + 2 g TXA in local, 15 mg/kg IV-TXA, 2 g TXA in local, and 100 ml IV, respectively. The results of total blood loss (TBL), maximum hemoglobin drop, the transfusion rate, and the number of allogeneic blood units were compared. Deep venous thrombosis (DVT) and pulmonary embolism (PE) events were monitored and recorded.

**Results:**

The TBL was 635.49 ± 143.60, 892.62 ± 166.85, 901.11 ± 186.25, and 1225.11 ± 186.25 mL for the combined group, the IV group, the topical group, and the control group, respectively (*p* = 0.015, *p* = 0.001, respectively). The average maximum hemoglobin drop in the four above groups was 2.18 ± 0.24, 2.80 ± 0.37, 2.40 ± 0.64, and 3.40 ± 1.32 g/dL, respectively. No PE event was reported during the follow-up. Although asymptomatic DVT events were reported by 1, 2, and 2 patients in the combined group, topical group, and control group, respectively, there is no intergroup difference.

**Conclusions:**

The combined use of TXA effectively reduced the total blood loss and blood transfusion rate in patients aged over 60 scheduled for a 2-level lumbar fusion, without increasing the incidence of DVT and PE formation.

## Introduction

Patients diagnosed with degenerative lumbar spine diseases such as lumbar spinal stenosis, disc herniation, and lumbar spondylolisthesis are generally required lumbar spinal fusion surgery [1, 2]. Lumbar spinal fusion surgery procedures were usually processed with the decompression, instrumentation, correction, and fusion procedures step by step. These procedures lead to relatively long operation time, especially for multi-level lumbar fusion cases, which are accompanied by a large incision and considerable blood loss. It was reported the average blood loss in noninstrumented lumbar fusion surgery was 800 mL, while the total blood loss of instrumented fusions could reach up to 1517 mL [3]. The blood loss during the operation was because of the abundant blood supply in the spongy vertebrae and the fragile vascular wall [3].

Massive blood loss makes the patients suffer a higher risk of developing cardiopulmonary events, renal failure, and cerebral infarction, especially for aged patients (over 60 years old) [4–6]. Although allogeneic blood transfusions could prevent the patients from suffering the above life-threatening complications, however, blood transfusions are usually limited with blood supply, potential risk of immunologic reaction, and infectious disease transmission [7].

To minimize perioperative blood loss, hemostatic agents have been regularly used. For instance, tranexamic acid (TXA) has been used to treat or prevent excessive blood loss from major trauma, surgery, nosebleeds, and heavy menstruation. TXA reduces bleeding by reversibly blocking the lysine binding sites on plasminogen molecules and thus efficiently inhibits fibrinolysis and stabilizes blood clots [8, 9]. TXA has demonstrated its efficiency in minimizing blood loss and transfusion rates in artificial joint replacement surgeries and spine surgeries [10–12]. However, the results of using TXA to reduce blood loss in lumbar spinal fusion surgery have been conflicting. Some researchers have reported convincing results of using TXA to minimize blood loss. On the contrary, other researchers insist that we need more solid proof to confirm the TXA’s efficiency of reducing blood loss. Besides, whether using TXA increase deep vein thrombosis (DVT) and pulmonary embolism (PE) formation or not is still on debate [13], especially in aged patients. Some researchers suggested using TXA increased the risk of thrombosis formation because the aged population generally suffering a higher risk of thrombosis formation. On the contrary, other researchers suggested using TXA would not increase thrombosis formation as TXA could only stabilize formed clots. Therefore, it is necessary to conduct a study to clarify the safety of using TXA in aged patients (aged over 60) operated with a 2-level lumbar fusion, by evaluating the incidence of thrombosis formation and systematic complications (including cardiac infarction, stroke, and acute renal failure).

Besides, another question that remained to be answered is which administration strategy of TXA possesses the highest efficiency of reducing blood loss in lumbar spinal fusion surgery. Generally, THA is administered with intravenous injection, and the IV practice has been increasing to its convenience and safety. Data collected from different surgical departments demonstrated TXA given in IV injection could reduce blood transfusion rate by up to 38% in various surgeries [14, 15]. Meanwhile, other studies suggested that the local use of TXA demonstrated a similar level or even higher hemostatic efficacy compared to IV TXA [16–18]. They proposed TXA administrated in situ could reach the bleeding sites much faster and accumulate at the surgical area, thus reducing blood loss more efficiently. Recently, studies reported the combined use of IV TXA and topical TXA to possess the highest hemostatic efficacy in artificial joint replacement surgeries [19–21]. However, the efficiency of using different TXA administration strategies in aged patients (aged over 60) operated with a 2-level lumbar fusion remains inconclusive. Thus, patients aged over 60 diagnosed with degenerative lumbar spine diseases such as lumbar spinal stenosis, disc herniation, and lumbar spondylolisthesis scheduled for a 2-level lumbar fusion were recruited. The patients were randomized into four groups and received different TXA therapies, and the efficiency was compared.

We conducted the present study to evaluate the safety of using TXA in patients operated with a 2-level lumbar fusion and simultaneously compared the hemostatic efficacy presented by different TXA management strategies.

## Materials and methods

### Patients and grouping

**Fig. 1:**
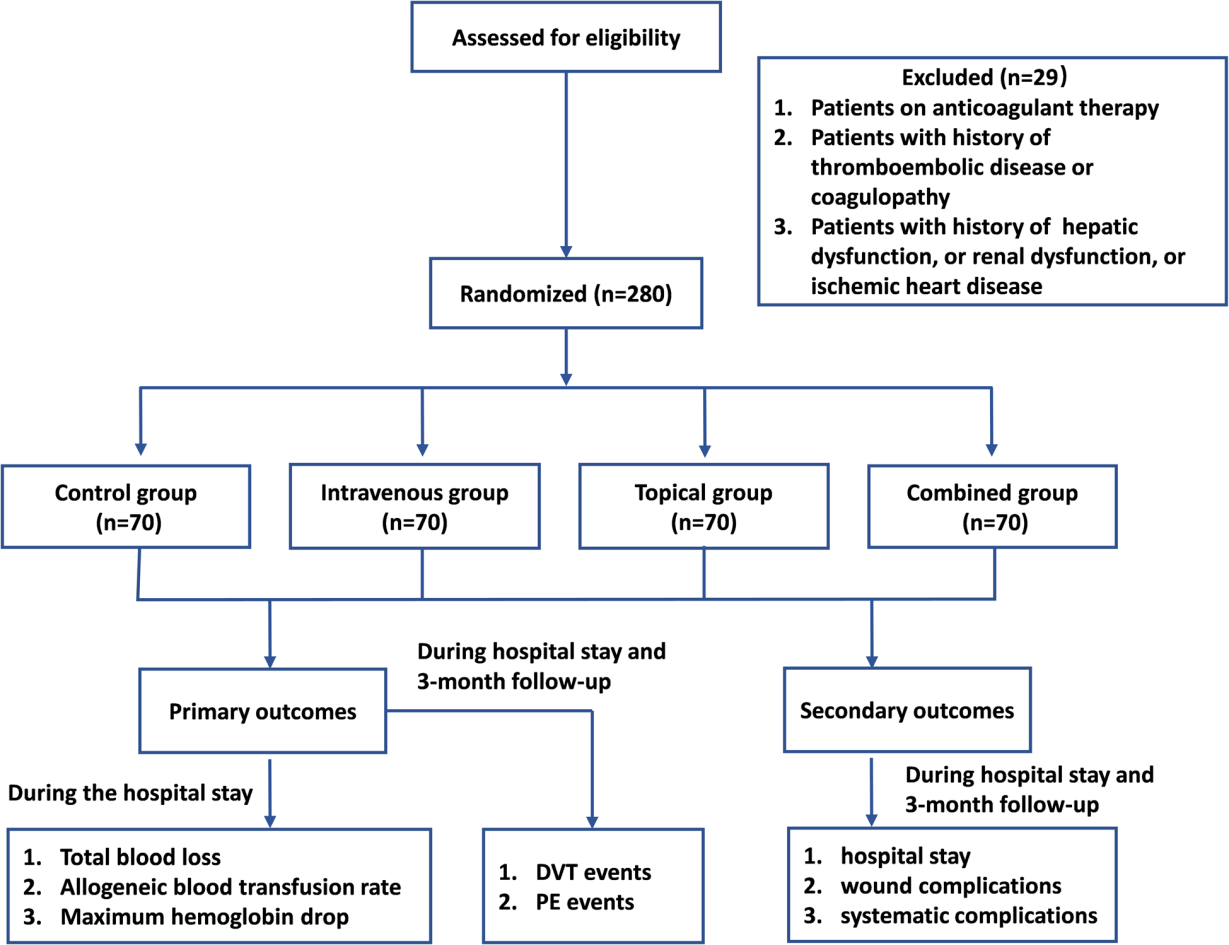
Flow chart of the whole study

From December 2015 to December 2019, 309 patients aged over 60 scheduled for a 2-level lumbar fusion were assessed for eligibility (Fig. 1). Patients diagnosed with a 2-level degenerative lumbar spine disease (spinal lumbar stenosis, disc herniation, or lumbar spondylolisthesis) were included in this study. We exclude the patients if they were receiving an ongoing anticoagulation therapy, complaining thromboembolic disease and coagulopathy history, and complaining hepatic or renal dysfunction or ischemic heart disease history. As a result, 280 patients were enrolled. The 280 patients were randomized into four groups, including a control group (group I), an intravenous (IV) TXA group, a topical TXA group, and a combined IV and topical TXA group IV. The current study was registered in the Chinese Clinical Trial Registry on March 1, 2015, which was then approved on March 13, 2015, with the reference number ChiCTR-IPR-15006088. The demographic data, preoperative hematologic results, and operation time of the recruited patients were presented in Fig. 1.

### Administration of TXA

Patients in the control group were given 100 ml IV saline. In the IV group, patients were given TXA at 15 mg/kg of body weight with a single intravenous dose 60 min before skin incision. As for patients included in the topical group, TXA saline (2 g in 20 mL) was injected into the incision by the drainage after the incision closure. Patients were given 2 g TXA in local and intravenous TXA simultaneously in the combined group.

### Surgical procedure and perioperative management

After general anesthesia, the patients were put in the prone position, a longitudinal incision is made in the midline of the low back over the spinal levels to be processed. Then, the fascia and muscle are divided and retracted to expose the posterior vertebral arches. A complete or partial laminectomy and foraminotomy (removal of bone spurs) were performed to decompress the nerves and remove the intervertebral disc. A cage filled with local autogenous bone was used for the fusion.

All the surgical procedures were conducted by the same surgical team in the same hospital. The spinal fixation system was supplied by the same manufacturer (Depuy Synthes, USA). The drainage was routinely clamped for 6 h and removed after 24 h. The patients were treated with a 30-min intermittent pneumatic compression pump every 8 h from the first-day post-surgery. Suspicious DVT symptoms, including pain, swelling, and tenderness in the lower limb, were monitored with attention before discharge. To monitor DVT formation, the patients were scanned with ultrasound on the day of discharge, 1- and 3-month follow-up. At the same time, ultrasound was performed immediately if any suspicious related to DVT symptoms complained. Computed tomography was taken to if PE needs to be ruled out.

### Blood transfusion guidelines

Necessary perioperative transfusion was performed in line with the transfusion management guidelines published by the Chinese Ministry of Health. Indications for a blood transfusion including low hemoglobin (< 70 g/L) and symptomatic anemia (including dizziness, shortness of breath heart failure, and reduced exercise tolerance).

### Outcome assessment

We recorded the primary outcomes and secondary outcomes to assess to the TXA therapy’s efficiency of reducing blood loss. The primary outcomes, including total blood loss (TBL), the allogeneic blood transfusion rate, maximum hemoglobin drop, and hematocrit change were compared to evaluate blood reducing efficiency. For the calculation of the total blood loss, we used a previously reported formula, in which the patient’s blood volume and Hb loss were the main variables [22–24]. The DVT and PE events were recorded to assess to the safety of using TXA. The secondary outcomes were composed of the during hospital stay, wound complications (including leakage, hematoma, and infection), and systematic complications (including cardiac infarction, stroke, and acute renal failure). In detail, we evaluated total blood loss, maximum hemoglobin drop, and allogeneic blood transfusion rate 3 days post operation. The length of hospital stay was recorded on the discharge day. In addition, we monitored the incidence of wound complications for 2 weeks after surgery. DVT events, PE events, and systematic complications were observed from day 1 after surgery till the end of the 3-month follow-up. During the 3-month follow-up, no case was lost.

### Statistical analysis

We used SPSS software (version 19.0) to analyze the statistical difference. For continuous variables, one-way ANOVA and Tukey’s post hoc test were applied to compare group differences. For qualitative comparative parameters, the Pearson chi-squared test or Fisher’s exact test was used to analyze, and a significant difference was confirmed when *P* < 0.05.

## Results

In the current study, from December 2015 to December 2019, 309 patients over 60 years old were conducted with a 2-level lumbar fusion surgery in our hospital. Based on the exclusion criteria, 29 patients were excluded. The included 280 patients were included randomized into 4 groups, as described above (Fig. 1). Demographic data including age, gender, height, body mass index (BMI), and preoperative laboratory parameters were recorded and compared (Table 1).

**Table 1:**
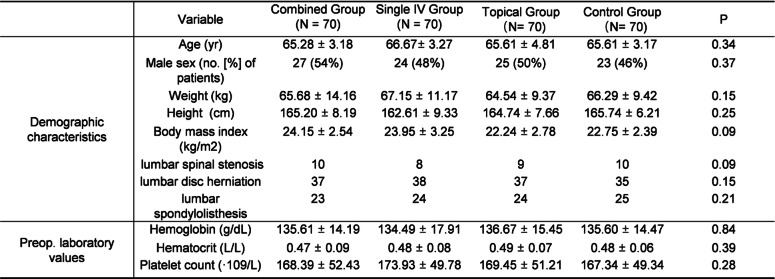
Baseline characteristics of the study population

*p* values indicate that were significantly different between the groups

The primary outcomes of the four groups were presented in Table 2. Specifically, TBL was 635.49 ± 143.60 mL in the combined group, which was considerably lower than the TBL of the IV group (892.62 ± 166.85 mL), the topical group (901.11 ± 186.25 mL), and the control group (1225.11 ± 186.25). Besides, the combined group also possessed a much smaller reduction in hemoglobin concentration and hematocrit. As shown in Table 2, the average hemoglobin drop of the combined, IV, topical, and control groups were 2.18 ± 0.24, 2.80 ± 0.37, 2.40 ± 0.64, and 3.40 ± 1.32 g/dL, respectively, demonstrating a significant intergroup difference (*p* < 0.05 for all). Following the blood transfusion guidelines, only 2 patients in the combined group had blood transfusions. However, 14 patients in the IV group, 12 patients in the topical group, and 34 patients in the control group required a blood transfusion, and transfusion rat indicated a significant intergroup difference. No PE was reported and detected during the whole process of follow-up. Asymptomatic DVT events were reported by 2 patients from the IV group, 2 patients from the topical group, and 1 patient from the combined group reported, respectively, while the intergroup difference was not statistically significant.

**Table 2:**
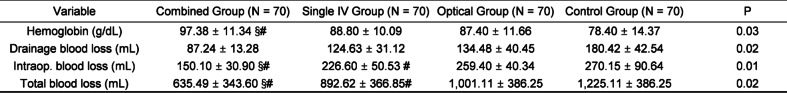
Primary outcomes regarding blood loss

The values are given as the mean standard deviation. *p* values indicate a significant difference among groups

The secondary outcomes of the four groups were demonstrated in Table 3. No systematic complications such as stroke, acute renal failure, or cardiac infarction had not complained during the hospital stay and follow-up. Although wound leakage was observed in 23 patients (4 patients in the combined group, 7 in the IV group, 5 in the topical group, and 7 in the control group), the differences were not statistically different. Other complications were not reported. The length of hospital stay and the cost of all the patients were compared and indicated no statistically significant differences.

**Table 3:**
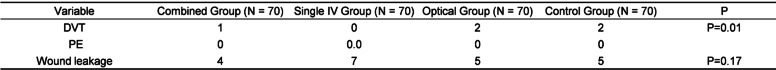
Outcomes regarding thrombosis formation and wound complications

The values are given as the mean and standard deviation. *p* values indicate a significant difference among groups

## Discussion

Lumbar spinal fusion surgery is a common procedure performed on patients diagnosed with degenerative lumbar spine diseases such as lumbar spinal stenosis, disc herniation, and lumbar spondylolisthesis of spine surgeries. Because of the rich blood supply in spongy vertebrae and removal of massive bone spurs to decompress the nerve system, average blood loss could reach up to 800–1517 mL in lumbar spinal fusion surgery [3]. Patients with massive blood loss are suffering a higher risk of cardiopulmonary events, renal failure, and cerebral infarction, especially for patients aged over 60. To reduce blood loss, the well-known anti fibrinolysis agent TXA has been widely used in THA, TKA, and spinal surgeries [10–12]. Intravenous TXA is still widely used, and topical use of TXA is becoming a routine procedure. Besides, the combined use of TXA has also been popularized [21]. However, the safety and hemostatic efficiency of the combined use of TXA in a 2-level lumbar fusion have been rarely reported, especially in patients over 60 years old. Therefore, we conducted the present study to investigate the hemostatic efficacy of different TXA management strategies on the blood loss in lumbar spinal fusion surgery. The demographic characteristics of the recruited patients were demonstrated in Table 1 parameters. As all the patients were randomly assigned, the results presented no demographic difference.

As shown in Table 2, compared to the other three groups, the results demonstrated that the combined used of TXA combined realized a much higher efficacy in reducing TBL and transfusion rates. Our results were consistent with previous studies, in which the combined use of TXA efficiently minimizes the blood loss in the TKA and THA surgeries [25–27]. The blood loss from lumbar spinal fusion surgery could be generally divided into an acute blood loss period (within 6–8 h post-surgery) and chronic blood loss period (within 24 h post-surgery). The accompanying fibrinolysis reaction in vitro could last for 24 h after the surgery [28]. In the current study, the IV tranexamic acid can maintain the serum antifibrinolytic activity 7 or 8 h, and the topical TXA could keep local antifibrinolytic activity up to around 18 h [29]. Therefore, the combined use of IV TXA and topical TXA could naturalize the fibrinolysis system in the first critical 24 h after surgery and thus efficiently reduce blood loss.

The combined therapy also possesses other advantages. Generally, the blood loss from lumbar spinal fusion surgery was due to incision hemorrhage and intramedullary hemorrhage. TXA administered intravenously could reach the incision site and diffuse broadly through microcirculation. Thus, the incision bleeding from the soft tissue could be efficiently inhibited by the preoperative TXA. In addition, the topical TXA could accumulate at the surgical area and directly target the intramedullary bleeding sites, which both could efficiently stabilize the blood clot and reduce hemorrhage. Herein, the incision hemorrhage and intramedullary hemorrhage could be efficiently reduced.

Inconsistent with the TBL results, patients in the combined group presented the lowest maximum hemoglobin drop compared to patients in the other three groups (Table 3). Specifically, the maximum hemoglobin drop was 2.18 ± 0.24, 2.80 ± 0.37, 2.40 ± 0.64, and 3.40 ± 1.32 g/dL for patients in the combined, IV, topical, and control group, respectively. The blood transfusion rate also presented a significant intergroup difference. In the combined group, only 2 patients required a blood transfusion. In contrast, 14 in the IV group, 12 in the topical group, and 34 patients in the control group were given blood transfusion based on the blood transfusion guideline.

As aged people are more likely to have hypertension, increased blood viscosity, and arteriosclerosis [30], which are risk factors of thrombosis formation. Thus, for patients aged over 60, some researchers are still worrying the use of TXA increases deep vein thrombosis and pulmonary embolism formation. However, at the end of the 3-month follow-up, no PE was reported. Asymptomatic DVT was recorded in different groups, but the intergroup difference was not statistically different (*p* = 0.623). These results further confirmed the safety of using TXA to reduce blood loss. TXA works by preventing active fibrinogen formation and inhibiting fibrinogen activation [31], thus, stopping fibrin hydrolysis, rather than stimulating fibrin formation and new thrombosis formation.

The limitations of the current study exist in the following aspects. First, the current study was conducted in a single center with a relative small sample size. The incidence of DVT or PE did not present intergroup differences here. However, a larger sample size might show different results. Second, although all the surgeries were conducted by the same surgical team in the same hospital, the individual differences of osteotomy and soft tissue release during the surgical process might have a potential influence on TBL, which were yet to be defined. Third, we only give a single dose of TXA to patients in the IV group in the current study. However, some researchers suggested repeated use of IV TXA may be more beneficial, which were adopted in the present study. Finally, the safety evaluation of using TXA in 2-level lumbar fusion surgeries in patients aged over 60 may need a longer follow-up, in which other adverse events may be found other than DVT and PE.

For patients aged over 60 operated with 2-level lumbar fusion surgeries, the current study demonstrates the combined use of IV TXA and topical TXA could efficiently reduce TBL and blood transfusion rate, without increasing the incidence of DVT and PE. The results of the current study will help reduce perioperative blood loss for patients processed with 2-level lumbar fusion surgeries.

## Conclusion

In conclusion, for patients aged over 60 operated with 2-level lumbar fusion surgeries, the combined use of TXA efficiently reduced TBL and blood transfusion rate, compared to IV use or topical administration alone. In addition, the combined use of TXA did not increase the incidence of venous thrombosis formation in the recruited 280 patients, and even they are prone to develop thrombus.

## Data Availability

The datasets during and/or analyzed during the current study available from the corresponding author on reasonable request.

## Abbreviations

TXA: Tranexamic acid
IV: Intravenous
TBL: Total blood loss
DVT: Deep venous thrombosis
PE: Pulmonary embolism
Hb: Hemoglobin
TBL: Total blood loss
BMI: Body mass index
